# Cystic fibrosis and Silver–Russell syndrome due to a partial maternal isodisomy of chromosome 7

**DOI:** 10.1002/ccr3.1061

**Published:** 2017-09-08

**Authors:** Lonneke C. Gerbrands, Eric G. Haarman, Margot A. Hankel, Martijn J. J. Finken

**Affiliations:** ^1^ Department of Pediatrics VU University Medical Center Amsterdam The Netherlands; ^2^ Department of Clinical Genetics VU University Medical Center Amsterdam The Netherlands

**Keywords:** Cystic fibrosis, growth disorders, Silver–Russell syndrome, small‐for‐gestational age, uniparental disomy

## Abstract

If an infant with cystic fibrosis exhibits failure to thrive, despite adequate disease management, Silver–Russell syndrome should be considered, given the locations of these conditions in the genome. However, an earlier clue to the diagnosis is small‐for‐gestational‐age birth.

## Introduction

Silver–Russell syndrome (SRS) is a clinically heterogeneous disorder characterized by intrauterine and postnatal growth restriction, hemihypoplasia, relative macrocephaly, and (usually subtle) craniofacial anomalies, such as frontal bossing. Most patients exhibit severe feeding difficulties 1, 2.

Several genetic defects can underlie SRS. Sixty percent of cases have hypomethylation of the imprinting control region 1 (ICR1) in chromosomal region 11p15 1, 2. Five to 10% of cases could be explained by maternal uniparental disomy of chromosome 7 (mUPD7), which is segmental in a minority of individuals 2.

Chromosomal region 7q31.2 harbors the cystic fibrosis (CF) transmembrane conductance regulator (CFTR) gene, which is mutated in CF patients. Consequently, the co‐existence of CF and SRS has been reported several times 3, 4, 5, 6, 7. Moreover, the first reports on UPD in humans occurred after the identification of maternally inherited homozygous mutations in the CFTR gene as a cause of CF 4, 5. This case report describes the clinical course of a patient with CF and mUDP7 of only a segment.

## Case History

A 3‐week‐old female newborn presented at our pediatric pulmonology department after she tested positive for the CFTR mutation F508del on neonatal screening. She was the second child of nonconsanguineous parents. Both her parents and her brother were healthy. She was born after an uneventful pregnancy at a gestational age of 36 weeks and 6 days. She had a birth weight of 2344 g (−1 SD score). Length was not measured at birth. On physical examination, we observed a relatively small child without apparent dysmorphic features or CF‐related symptomatology. Her length was 45 cm (−3.5 SD score according to postnatal norms, and −2.8 SD score according to intrauterine norms).

**Figure 1:**
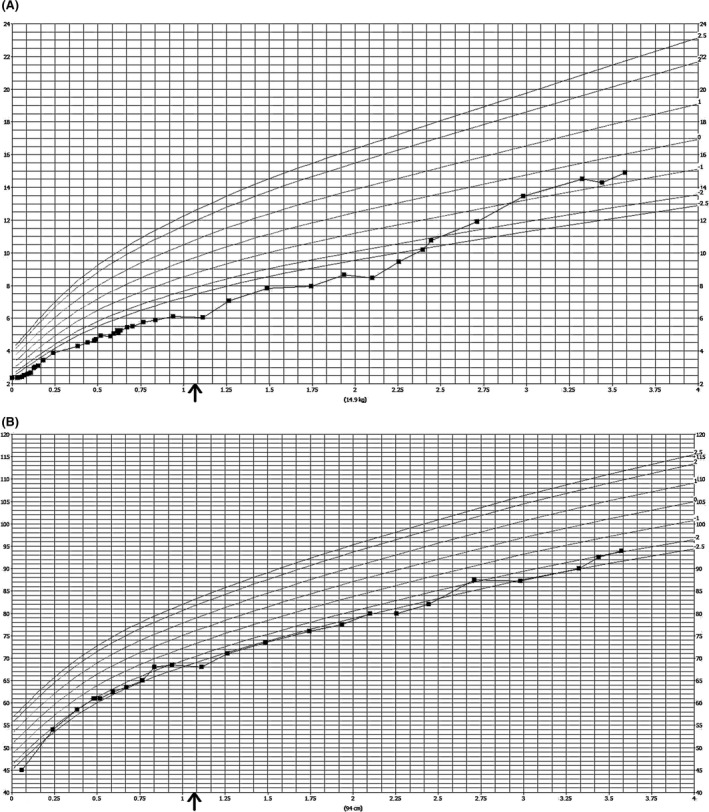
The arrows indicate the timing of percutaneous endoscopic gastrostomy tube placement. Growth curves of our patient. (A) Weight, *x*‐axis: age (years), *y*‐axis: weight (kg). (B) Length/height, *x*‐axis: age (years), *y*‐axis: length/height (cm).

A positive sweat test and a line probe assay confirmed the diagnosis of CF. Subsequently, she commenced with pancreatic enzymes, fat‐soluble vitamins, and salt supplementation. During the first months after initial presentation, she caught up in weight and length. From the age of 3 months, her weight started to lag behind, whereas her length remained stable (Fig. 1A and B). This pattern was accompanied by a poor appetite, with an estimated caloric intake of 700 kcal/day. Subsequently, she switched to an energy‐enriched diet, and the dose of pancreatic enzymes was increased to 10,000 IU per kg body weight per day. To further improve fat digestion, a proton‐pump inhibitor was added. Tube feeding was initiated at 9 months.

Due to persistent growth failure and clinical suspicion of gastroesophageal reflux disease (GERD), treatment with thickened feeds and a dopamine antagonist was initiated at 12 months. The deflection of the growth curve continued and, eventually, she lost weight (Fig. 1A). Therefore, at age 13 months, a percutaneous endoscopic gastrostomy (PEG) tube was placed.

At that time, segregation analysis revealed that the mother exclusively carried the CFTR F508del mutation. No paternal pathogenic mutation in or deletion of the CFTR gene was found. Moreover, the possibility of nonpaternity was excluded. Therefore, a subsequent UPD analysis was performed using a PCR‐based analysis with 11 variable number tandem repeat markers on chromosome 7. This test showed homozygosity for seven consecutive markers and maternal‐only inheritance for three markers, indicative of partial mUPD7 that covered at least chromosomal region 7q31‐q34 (Table 1). She was subsequently referred to a pediatric endocrinologist, who confirmed clinical suspicion of SRS 8. She was likely born small‐for‐gestational age (SGA) for length and exhibited four of the five other criteria for SRS: postnatal growth restriction, relative macrocephaly, frontal bossing, and feeding difficulties. Body asymmetry was lacking. Thus, she fulfilled the criteria for SRS.

**Table 1:** VNTR marker analysis of chromosome 7

Marker	Position	Patient	Father	Mother	Informative	Conclusion
D7S2201	7p22.1	104/108	104/108	104/108	No	–
D7S1802	7p15.3	186/183	186/183	178/183	Yes	No UPD
D7S1808	7p15.1	258/255	258/251	269/255	Yes	No UPD
D7S1830	7p12.1	201/214	201/223	214/214	Yes	No UPD
D7S2847	7q31.31	194/194	179/190	194/194	Yes	mUPD
D7S1804	7q32.3	262/262	266/273	269/262	Yes	mUPD
D7S1824	7q34	189/189	165/185	185/189	Yes	mUPD
D7S2195	7q35	284/284	284/291	287/284	No	–
D7S1805	7q36.1	213/213	213/209	196/213	No	–
D7S550	7q36.3	191/191	191/187	189/191	No	–
D7S559	7q36.3	195/195	195/199	201/195	No	–

VNTR, variable number tandem repeat.

After PEG tube placement, a gradual improvement in weight was observed, whereas length remained stable between the −2 and −2.5 SD lines (Fig. 1A and B). Our patient is now 4 years of age. Her height is slightly below the −2 SD line according to Dutch norms (Fig. 1B) and at the +1.5 SD line for girls with SRS. Her weight is normal (Fig. 1A). There is a slight delay in her gross motor development. However, her fine motor skills and speech development are age appropriate.

## Discussion

We describe a patient who suffered from both CF and SRS due to a partial mUPD7. Although there was maternal isodisomy of only a segment of chromosome 7, which covered at least region 7q31‐q34, our patient exhibited similar SRS‐related symptoms as other patients with mUPD7, including intrauterine and postnatal growth restriction, relative macrocephaly, frontal bossing, and feeding difficulties. However, hemihypoplasia, which is a hallmark of SRS, was lacking in our patient. This finding is compatible with previous reports demonstrating that mUPD7 is associated with a milder SRS phenotype, including less hemihypoplasia, than in cases of ICR1 hypomethylation 1, 2.

Our patient with CF and SRS due to mUPD of only a segment of chromosome 7 had a clinical presentation that was no different from previously reported patients with CF and SRS due to mUPD7. It is unclear whether segmental mUPD7 was excluded in the previously reported cases 4, 5, 6, 7. Given that more recent evidence suggests that segmental mUPD7 is only noted in a minority of patients with SRS, with 7q31‐qter being the most likely candidate region 9, it is conceivable that we have reported on the first patient to date with CF and SRS due to partial mUPD7.

The case presented in this report illustrates that SRS is accompanied by severe feeding difficulties, including poor appetite, GERD, and failure to thrive. This finding is of paramount importance in CF, where a lower weight or lower body mass index is associated with a more rapid deterioration in pulmonary function and increased mortality 10. Therefore, the achievement and maintenance of an optimal nutritional status is one of the mainstays in CF treatment 11, 12. Given the risks associated with a suboptimal nutritional status in CF, we would like to comment briefly on current practice guidelines. First, the CF Foundation consensus document for the management of infants with CF does not consider SRS, although rare, as a possibility in the work‐up of patients with insufficient weight gain 11. Although SRS is characterized by a set of relatively mild dysmorphic features that are easily overlooked at examination, an SGA birth should alert clinicians to this possibility. Second, early initiation of tube feeding is warranted in patients with both conditions to avoid a suboptimal nutritional status and weight loss. However, this must be balanced against recent recommendations for patients with SRS, stating that caution should be exercised with nutritional management 8. The dietary requirements of patients with SRS are typically lower given their relatively low muscle mass. Rapid weight gain is associated with increases in fat content and long‐term cardiometabolic risks 8. Third, growth hormone treatment is known for its anabolic effects and can be considered in children born SGA who remain short, including those with SRS 8. However, its efficacy and safety in patients such as ours are unclear.

## Authorship

LCG, EGH, MAH, and MJJF: performed the data acquisition and interpretation. LCG: prepared the figures. MAH: prepared the table. LCG and MJJF: drafted the manuscript. LCG, EGH, MAH, and MJJF: edited, reviewed, and approved the final version of the manuscript.

## Informed Consent

Obtained from the parents.

## Conflict of Interest

None declared.
